# The adaptive capacity of smallholder mixed-farming systems to the impact of climate change: The case of KwaZulu-Natal in South Africa

**DOI:** 10.4102/jamba.v9i1.469

**Published:** 2017-11-27

**Authors:** Nonhlanzeko N. Mthembu, Elliot M. Zwane

**Affiliations:** 1 South African Department of Agriculture, Forestry and Fisheries, South Africa; 2 Department of Economics and Animal Science, Centre for Rural Community Empowerment, University of Limpopo, South Africa

## Abstract

Climate change poses a serious threat to efforts by developing countries to ensure food security and poverty reduction. The National Development goals of South Africa envisage the agricultural sector as a key driver for job creation and economic growth. This article seeks to investigate the adaptive capacity of the Ncunjane farming community in Msinga, KwaZulu-Natal in response to drought spells of 2010 and 2014. This article draws on data collected using both qualitative and quantitative methods in 2011 and later in 2015 with the data analysed through the Statistical Package for Social Science to determine significant correlations between variables. Analysis of the vulnerability and adaptive capacity is performed using conceptual framework. This study found that both smallholder farmers who engaged in livestock and crop production have experienced high cattle mortalities and stagnant crop productivity, which in turn put pressure on already constrained disposable household income because of increased food costs and agricultural input costs, particularly supplementary animal feed. Cattle owners were more vulnerable to drought because of poor risk management and thus became highly dependent on government to provide drought relief. Application for government drought relief was found not to be effective in cases of large herds of cattle. Variability of rainfall and prolonged heat spells has a significant impact on the sustainability of smallholder mixed-farming systems, leaving agriculture as a highly questionable form of livelihood for rural farming communities such as Msinga. The article recommends strengthened institutional mechanisms so that stakeholders should play a more meaningful role within provincial and local agriculture in leveraging government support but places emphasis on the adoption of innovative strategies that can potentially yield significantly resilient smallholder mixed-farming systems in the wake of climate variability.

## Introduction

Climate change is generally regarded as a threat to efforts by developing countries to ensure food security and poverty reduction (Department of Environmental Affairs and Tourism [DEAT] 2004). The OECD (2012) acknowledged that climate change is exacerbating the challenges faced by the agriculture sector in that climate change–induced increases in temperatures, rainfall variation and the frequency and intensity of extreme weather events are adding to pressure on the global agriculture system – which is already struggling to respond to rising demands for food and renewable energy. The changing climate is also contributing to resource problems beyond food security, such as water scarcity, pollution and soil degradation. Changes in climate have also been found to have adverse effects on the economy at large. The DARA and Climate Vulnerable Forum (2012) released a study, which shows a 23% reduction in global gross domestic product (GDP) by the year 2100 as a result of climate change with an estimated 1.6% loss annually. Statistics published much earlier also show similar losses in GDP with a predicted decline of the GDP to just 1.5% with net crop revenues falling by 90% by 2100 in South Africa (Hassan 2006).

The South African agricultural sector is by no means immune to the impacts of climate change; rainfall is erratic coupled with seasonal variation and high evapotranspiration, causing a reduction in both surface and groundwater resources (DEAT 2004). Hassan (2006) and Nkomo, Nyong and Kulinda (2006) found that South Africa will experience a 0.13 °C rise in day temperatures with dry land and smallholder farmers being mostly affected as compared to irrigation and large-scale or commercial farmers. It was also predicted that there would be an extensive reduction of rainfall that is said to likely be in the range of 5% – 10% in the summer rainfall region (DEAT 2004).

Such is the case in the KwaZulu-Natal (KZN) Province where extreme drought conditions were experienced by the farming fraternity during the growing season of 2014 through to late 2015, which had a more pronounced effect on the small-scale enterprises characterised by limited resources. Smallholder producers raised concerns over exorbitant agricultural input costs and the protracted period of low returns on production. The worse impact was on dry land conditions with smallholder producers experiencing increased crop losses and high cattle mortality rates because of a lack of adequate feed and water. In response, the KZN Provincial Department of Agriculture and Rural Development implemented a Drought Relief Plan to assist farmers by subsidising animal feed while the Department of Water and Sanitation provided much needed water to communities (South African Government 2015).

This research article aims to firstly present findings on immediate mitigation measures taken by the Ncunjane farming community in Msinga, KZN in response to drought in 2010 and 2014. This article will also investigate the community’s adaptive capacity to climate variability impacts through analysis of their responses using the conceptual framework of Intergovernmental Panel on Climate Change (IPCC) (2014). Furthermore, the article seeks:

- to critically assess different strategies and mitigation measures towards enhancement of smallholder producers’ adaption to climate variability.

## Theoretical background

Conflicting concepts on definitions of key terminology used within the climate change discourse have surfaced in most published material, namely vulnerability, adaptability, adaptive capacity and resilience. These key search words result in more accurate search results when conducting literature reviews on climate change as they generate a mountain of journal articles, occasional paper series, books and newspaper articles. Two theoretical frameworks begin to emerge from analysis of this literature, namely one being the different entry contextual points of analysis used to derive definitions of climate change and the other being the design of research objectives sought in addressing climate change impact. This leads to asking different questions and application of different data collection and analysis tools and methods to arrive at hypothesised or presumed answers.

### Conceptual frameworks on adaption to climate change

The IPCC’s Climate Change 2007 and 2014 Synthesis Reports emerge as the dominant documentation and publication on climate change; hence, this article largely depended on this work as a point of reference. Methodological theories on deriving a better understanding of climate variability impact on society including societies’ immediate and long-term responses to this global phenomenon are well documented by the IPCC. This article seeks to dissect the vulnerability and adaptive capacity of the Ncunjane community to climate change impact using two conceptual frameworks advanced by the IPCC that are based on the following approaches:

- Top-down approach, which focuses on physical vulnerability and assumes that people and ecosystems are vulnerable to climate change.
- Bottom-up approach, which focuses on social vulnerability and looks at the local scale on top of which climate change occurs. Table 1 illustrates these conceptual frameworks.

**TABLE 1:** Conceptualisation of climate change impact and vulnerability.

Context	Climate change impacts perspective	Vulnerability perspective
Root problem	Climate change	Social vulnerability
Policy context	Climate change mitigation, compensation, technical adaptation	Social adaptation, sustainable development
Illustrative policy questions	What are the benefits of climate change mitigation	How can the vulnerability of societies to climatic hazards be reduced
Illustrative research questions	What are the expected net impacts of climate change in different groups	Why are some groups most affected by climatic hazards than others
Vulnerability and adaptive capacity	Adaptive capacity determines vulnerability	Vulnerability determines adaptive capacity
Reference for adaptive capacity	Adaptation to future climate change	Adaptation to current climate variability
Starting point of analysis	Scenarios of future climate change	Current vulnerability to climatic variability
Analytical function	Descriptive, positivist	Explanatory, normative
Main discipline	Natural science	Social science
Meaning of vulnerability	Expected net damage for a given level of global climate change	Susceptibility to climate change and variability as determined by socio-economic factors
Vulnerability approach	Integrated, risk hazard	Political economy
Reference	IPCC (2001)	Adger (1999)

*Source*: Adapted from Fussel, H.-M., 2007, ‘Adaptation planning for climate change: Concepts, assessment approaches, and key lessons’, *Journal of Sustainability Science* 2(2), 265–275. https://doi.org/10.1007/s11625-007-0032-y

### Problem to be investigated

Small-scale farming systems in South Africa often include a mix of crop and livestock production and serve multiple households functions and objectives, such as the provision of food (meat and milk), manure and being sold to get immediate cash. These farming systems are commonly characterised by low production, poor access to productive assets such as infrastructure and credit (Sebopetji & Belete 2009) and structural constraints such as high transaction costs and weak links to agro-value chains (Kirsten & Van Zyl 1998:551; Lahiff & Cousins 2005:127). Operating under a limited-resource base puts pressure on small-scale production systems; hence, with the addition of climate variability factors, the sustainability of these production systems comes under strain. Drought as a result of the reduction in rainfall during the summer rainfall season was experienced by the Ncunjane farming community in 2010 and again in 2014–2015. In both cases, households experienced high livestock mortality rates and crop failure. It is the researchers’ observation that throughout these incidences, this community was vulnerable and became highly dependent on government support interventions, which often were ineffective given the large numbers of livestock needing relief. Stock owners did not have risk mitigation strategies in place and lacked resources to either mitigate or adapt to climate variability. The poorly resourced farming households to whom agriculture is a major livelihood source are hard hit by droughts and take longer to recover. Hence, this article seeks to investigate innovative strategies for these households to adapt with climate variability towards more resilient and sustainable production systems.

It is the writers’ understanding though that the illustrated perspectives presented in Table 1 are not cast in stone and should, with more work conducted in the field, be evaluated and refined accordingly if research on climate change is to have a greater impact on strengthening policy development and strategies aimed at addressing complexities of climate change impact experienced by farming communities. Over and above this, the writers agree with the IPCC’s Forth Synthesis Report (IPCC 2007) that the key is to find multidisciplinary conceptual frameworks, which fuse social, ecological and financial perspectives to assess and address risks associated with climate change.

## Research methodology

The methodology adopted for the paper consisted of the literature review based on theoretical key search words used in the climate change discourse to conduct a thorough Internet search on Google Scholar for publications. The focus was on literature documenting climate change impact, community responses and adaptation strategies published between 2006 and 2016. Literature prior to 2006 was not reviewed as this was covered by the IPCC Forth Synthesis Report (IPCC 2007). The report was used as the reference point to conduct secondary research of more journal articles. Data collection further focused on articles providing sound statistical data and figures on climate change as well as IPCC extensive publications on the impact of climate change on the agricultural sector in Africa (Berrang-Ford, Ford & Paterson 2011). Other journal articles were consulted to supplement the IPCC knowledge hub because IPCC produces a comprehensive assessment on the state of knowledge on climate change approximately every 5 years. Empirical data were collected based on rural small-scale mixed-farming systems in Ncunjane, Msinga in the KZN Province in 2011 (Mthembu 2013). Quantitative data were collected using structured questionnaires and measuring of field plots, which were analysed using Statistical Package for Social Science (SPSS) to determine significant correlations between variables. Hence, data on household profiles; household composition, sources of incomes and farming activities are already available. Qualitative data were collected using focus groups and in-depth interviews with crop producers and livestock owners.

## Results and discussion

The results of the study are presented in this section, namely the effects of variability in rainfall on these production systems and the immediate control measures put in place by households including interventions from the local and provincial government. Evidence of the effects of the recent drought in the study area is also presented (Vanderhaeghen & Horny 2016).

### Socio-economic status of households in Ncunjane

Ncunjane is located in rural Msinga in the KZN Province and is characterised by multigenerational former labour tenant families living in large compound homesteads where both livestock and crop production is the mainstay on land accessed through the land reform programme in 2000. The data collected in 2011 were based on 22 of the 24 households in the Ncunjane community interviewed. Table 2 indicates that household sizes range from 4 to 21 members with adult ages ranging between 18 and 93 years (Mthembu 2013).

**TABLE 2:** Demographic features of households in Ncunjane (*n* = 22).

Demographic profile	Mean	Median	Range	Proportions
Household size	9.27	8	4–21	72.6% have 4–10 members; 27.1% have 11–21 members
Generations in household	2.45	3	1–3	Three households have 1 generations; 6 have two generations; 13 have three generations
Age of adult members	38.91	34	18–93	-
Sex of adult members	-	-	-	There are 64 (59.8%) women and 43 (40.2%) men from a total of 107 adults in the population sample of 204

*Source*: Mthembu, N.N., 2013, ‘An investigation of characteristics of mixed farming systems: The case of labour tenant communities of Ncunjane and Nkaseni in Msinga, KwaZulu-Natal’, Submitted in partial fulfilment of requirements of the Master Degree of Philosophy in Land and Agrarian Studies, Institute for Land and Agrarian Studies (PLAAS), Bellville

From a socio-economic perspective, social grants are the main source of income (33% from child grants and 11.43% from old-age grants) among households with permanent jobs contributing 10.48% to household disposable income (Mthembu 2013). Agricultural sales comprise vegetables and grains (maize and dry beans) sold mostly by women and livestock (cattle and goats) sold mainly by men and contributed 9.52% to the household income and was the fourth major income source as seen in Table 3. Household expenditure indicates a high investment in domestic and electronic assets (53.77%) and in agricultural assets (42.65%). The high reinvestment into agricultural production is expected as 78% of the 22 households’ surveyed kept cattle, which included a noticeable four households entering into cattle production during the year 2011–2012 after the 2010 drought indicating the significant contribution derived from agriculture for this farming community (Mthembu 2013).

**TABLE 3:** Income sources of adult household members in Ncunjane.

Household income sources	Total income sources (*n* = 105 in Ncunjane)					
	Male		Female		All adults	
	*n*	%	*n*	%	*n*	%
Employee in permanent job	5	16.67	6	8.00	11	**10.48**
Employee in temporary, contract job	4	13.33	4	5.33	8	7.62
Do casual employee work	1	3.33	4	5.33	5	4.76
Farming activities on homestead’s land that results in cash income	5	16.67	5	6.67	10	**9.52**
Self-employed in non-agricultural own or family income-earning activity without employees	0	0.00	2	2.67	2	1.90
Self-employed in non-agricultural own or family income-earning activity with employees	7	23.33	3	4.00	10	**9.52**
Work on income-generating project	0	0.00	0	0.00	0	0.00
Not employed and looking for work	1	3.33	1	1.33	2	1.90
Not employed and not looking for work	1	3.33	0	0.00	1	0.95
Old-age grant from government	3	10.00	9	12.00	12	**11.43**
Pension from private employer	0	0.00	0	0.00	0	0.00
Disability grant	2	6.67	0	0.00	2	1.90
Child support grant	0	0.00	35	46.67	35	**33.33**
Remittances in cash	1	3.33	6	8.00	7	6.67
Remittances in kind (e.g. food, clothes, etc.)	0	0.00	0	0.00	0	0.00
Others – specify	0	0.00	0	0.00	0	0.00
**Total**	**30**	**100**	**75**	**100**	**105**	**100**

*Source*: Mthembu, N.N., 2013, ‘An investigation of characteristics of mixed farming systems: The case of labour tenant communities of Ncunjane and Nkaseni in Msinga, KwaZulu-Natal’, Submitted in partial fulfilment of requirements of the Master Degree of Philosophy in Land and Agrarian Studies, Institute for Land and Agrarian Studies (PLAAS), Bellville

The bolded values are to present the main sources of income as a percentage of the total value and are referred to in the analysis of the table in the paragraph to follow.

Agriculture serves as a dominant socio-economic and cultural factor in Ncunjane and evidence. Table 4 supports this notion as the mean cattle ownership is 15.68 and that of goat ownership is 44.33 coupled with varying land used for cropping ranging from 0.025 ha to 0.08 ha for garden plots and from 0.13 ha to 2.03 ha for field plots (Mthembu 2013). Cattle are regarded by most people as the most important livestock species, although not all people do keep cattle. They are animals that traditionally belong to men. Cattle are used for draught, for *lobola*, for ceremonial slaughter, for hides that are used to make traditional clothing, for meat and for sales (Cousins 2011:12).

**TABLE 4:** Cattle ownership in 2012 at Ncunjane (*n* = 22).

Variables	Cattle data	Cattle ownership by households	Male heads	Female heads
Mean	15.68	0 cattle	5	0
Median	10	1–25 cattle	5	5
Sum	483	26–50 cattle	4	0
Minimum	0	51–75 cattle	1	2
Maximum	63	> 76 cattle	0	0
Range	0–63	-	-	-

*Source*: Mthembu, N.N., 2013, ‘An investigation of characteristics of mixed farming systems: The case of labour tenant communities of Ncunjane and Nkaseni in Msinga, KwaZulu-Natal’, Submitted in partial fulfilment of requirements of the Master Degree of Philosophy in Land and Agrarian Studies, Institute for Land and Agrarian Studies (PLAAS), Bellville

### Effects of drought on the communities

The community of Ncunjane was affected by the drought of 2014–2015 and some of the effects were documented in an article by Vanderhaeghen and Hornby (2016) in the Daily Maverick local newspaper, which painted a gruesome picture of cattle carcases covering the arid, dusty, cracked landscape without any green sprout in sight. On one occasion, one household lost 40 of its 70-cattle herd, which constitutes an extremely high loss (57%). Similar high rates of cattle and goat mortalities were reported in the 2010 drought with households recalling that they had lost up to five cattle overnight, with many being pregnant at the time of death (Mthembu 2013).

Further effects of the drought spells were also captured by Mthembu (2013) as a result of the drought incidence in 2010. The cause of the hardships in the community was climate change; this was so because the area experienced very low rainfall over the October–December 2010 period with an extreme increase in day temperatures causing a high rate of evapotranspiration of the nearly non-existent surface water. Consequently, the main water sources, the Tugela and Mooi rivers, dried up, consequently annual dry land cropping of black kernel maize and sorghum on large fields and vegetable production on small plots was postponed, thus forcing households to surrender being producers of the staple maize crop to consumers who have to succumb to high-priced maize meal bags. A 50-kg or 80-kg bag of maize meal was estimated at R200 and R460, respectively, before the drought. However, the prices have since increased to R290 for a 50-kg bag and up to R600 for an 80-kg bag of maize meal (Mthembu 2013) in 2011.

The drought also indirectly affected the socio-economic status of the community; for instance, it was reported that the local borehole water pump was not operational because of a lack of diesel. As a result, there was very limited water both for domestic and agricultural use (Vanderhaeghen & Horny 2016). Given the low economic profiles of households, which are highly dependent on social grants for a stable income, such harsh circumstances tend to increase the vulnerability of households as a higher proportion of the disposable income gets channelled to procuring food. This reprioritisation of the available household budget is necessary to ensure household food security and avoid malnutrition of children.

### Dependency upon government support during drought

The study revealed little or no independency by livestock farmers to cope with the distress of the drought incidence of 2014–2015, acknowledging that the opportunity for putting in place effective coping mechanisms was presented during the initial drought in 2010. Mthembu (2013) observed that when faced with a shortage of grazing, cattle would feed on maize stalks in surrounding crop fields, and in worse cases cattle owners would seek refuge for their cattle in a neighbouring commercial farm where there is plenty grazing. However, the latter strategy requires cattle owners to pay R50 per animal per day, which for households with large herds of cattle and limited income was simply not feasible and therefore unsustainable.

Fast-forward to 2015, no proper immediate risk management plan was in place to assist during drought by the cattle owners. Vanderhaeghen and Horny (2016) observed that cattle owners having more than 10 cattle recalled partaking in a cumbersome application process for accessing government drought relief support in the form of supplementary animal feed (i.e. molasses, maize and grass). Apart from the long queues and strict requirements that were put in place, they had to produce validation documents to authenticate their cattle ownership status. It was found that not all cattle owners had their own cattle dipping records or brand mark certificates. As a result, some owners gave up trying to meet such requirements and forfeited the much needed support. This led to devastating cattle losses. This indicates poor planning and management among cattle owners and a high dependency on government support to help cushion the impact of climate change. Further analysis of the community’s vulnerability and adaptive capacity is presented in Table 5 and is based on research findings of Mthembu (2013).

**TABLE 5:** Vulnerability and adaptive capacity matrix.

Theoretical Framework	Adaptive capacity	Vulnerability
Adaptive capacity determines vulnerability	Sell off cattle at the onset of changes in climate and use money in non-farm investments or keep it for reinjection into livestock production upon return of favourable conditions.	Reduced cattle numbers perceived as diminishing socio-economic status among cattle owners as keeping large herds is associated with a sign of wealth. Cattle sold at the expense of other uses of cattle, for instance, as a food source (milk and meat), cultural slaughter, and so forth, thus increasing household expenditure on food.
	Invest more on goat production as goats prove to be more resilient under drought conditions.	Capita per goat is greatly reduced compared to that generated by an ox and therefore not as profitable as cattle production. Households have to consider the risks of cattle production over the economic value derived from goat production.
	Procure hay bales as supplementary feed when there is a lack of grass during drought.	Not feasible for rural households as an immediate control measure as hay is too expensive. Limited supply and high demand because of large cattle numbers per household.
	Use of the neighbouring game farm to access grass and water for cattle during winder and drought conditions.	Not sustainable as stockowners have to pay R50 per animal per day, considering large herds and limited incomes. Other expenses are incurred from this strategy when cattle feed illegally and are impounded for illegal trespassing, with more cattle dying or unable to recover during impounding or after release resulting in fruitless expenditure.
	Halt annual dry land cropping on large fields and limit to small vegetable production.	Forces prolonged migration from producer to consumer of staple food crops. The high costs of maize meal again further limits household’s purchasing power (social grants are the main income source).
Vulnerability determines adaptive capacity	Sell healthy cattle and generate income for procuring inputs for remaining stock (i.e. supplementary feed, diesel for borehole pump so cattle have access to minimum water needs). ALTERNATIVELY: Sell off herd and use capital injection into other business or invest appropriately for future use (option to return to livestock production when climate is favourable).	High cattle mortality rates especially heifers and cattle in calving.
	Goats are beneficial: Goats are resilient under drought. Self-herding requiring less human management. Feed on large variety of bush species so food is readily available. Requires less water per day than cattle. Are in high demand in informal markets in rural areas because they are important in cultural rituals. Present agro-processing potential (milk, cheese, skin hides).	Decision to forego cattle for goat production may present discomfort because of fear of the unknown possibilities and even failures.
	Cattle owners need capacity development in: budget planning risk management planning and scenario planning to cater for extreme cases.	Use of savings for executing mitigation measures is not economically feasible.
	Cattle owners need capacity development in: rotational grazing camps savings account for implement contingencies culling herd sizes plant grasses with high water content and obtain feed with low salt content.	Use of savings for executing mitigation measures is not economically feasible.
	Cattle owners need capacity development in: invest in crop cultivars (drought resistant; short growing season) prioritise available water for irrigation purposes invest in climate-smart irrigation innovation (water harvesting and conservation practices and infrastructure) restrict scale of production alternative in-house production systems (greenhouse, hydroponics).	Poor households at the mercy of rising food prices.

*Source*: Mthembu, N.N., 2013, ‘An investigation of characteristics of mixed farming systems: The case of labour tenant communities of Ncunjane and Nkaseni in Msinga, KwaZulu-Natal’, Submitted in partial fulfilment of requirements of the Master Degree of Philosophy in Land and Agrarian Studies, Institute for Land and Agrarian Studies (PLAAS), Bellville

### Adaptation to current and future climate variability

**FIGURE 1:**
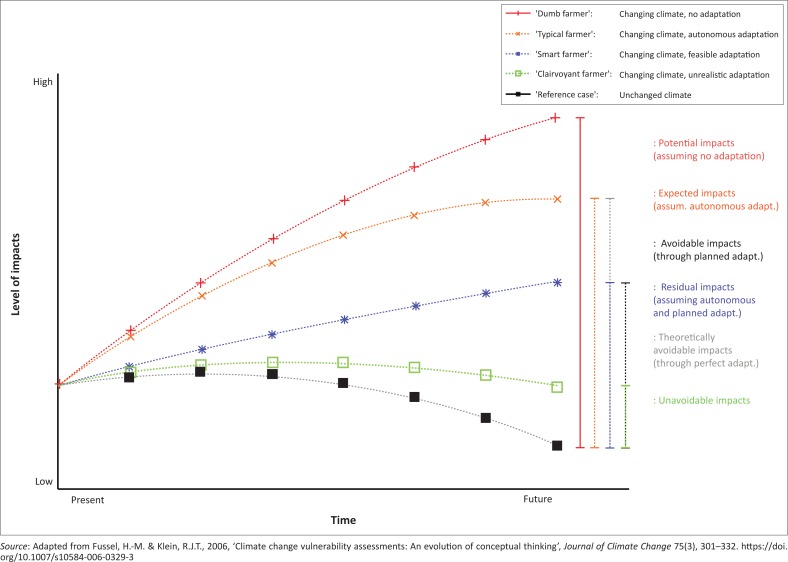
Climate change and adaptive capacity of farmers.

The work on climate change impacts and adaptation conducted by Fussel and Klein (2006) produced useful reference points for analysing adaptive capacity of households in the study area. The notion is that the greater the adaptive capacity of the farmer, the less climate change impact will be experienced versus farmers with very little or lack of adaptive capacity (Figure 1).

### Principles of adaptation

Fussel and Klein (2006) identified some of the principles about adaptation. They should be guided by the following:

- Need arises from extreme events rather than average climatic conditions.
- Vulnerability resulting from extreme events requires assessing both natural climatic vulnerability and anthropogenic climate change.
- There is a fine line between reactive and proactive adaptation in practice.
- Adaptation to climate change is a continuous process.

Access to reliable, timely and accurate information about future changes in climate can help reduce the total costs of adaptation. Addressing vulnerability to current changes in climate on the other hand requires access to adequate resources, over and above information, at the farmer’s disposal to aid in effective and efficient mitigation planning and execution.

### Factors increasing vulnerability

The lack of information and early warning systems including regular weather reports leads to poor risk assessment and mitigation, therefore increasing exposure to risks, some of which are avoidable (Fussel & Klein 2006). In Ncunjane, two major factors were found to increase vulnerability. Firstly, crop farming systems are exposed to climatic variability with producers having minimal control over the effects of climate change especially under dry land agriculture. Secondly farming households in Ncunjane have a high dependency on external sources of support for their agricultural inputs like seeds, fertiliser, tractor services, and pesticides including drought relief support for livestock. Should these support services be delayed or not provided, households are left with very limited or no options to sustain their farming systems.

### Factors increasing adaptability

Fussel and Klein (2006) note that to shift farmer’s perceptions and perspectives from traditional crop and livestock production practices do not come easy but prove to be a worthwhile exercise as the future of smallholder farming systems is threatened by changing climate. There is opportunity to assist farming households increase their adaptive capacity through the gradual introduction of critical decision-making tools and systems such as putting in place functional early warning systems coupled with proper knowledge and information management systems. The implementation of basic agribusiness management principles and risk mitigation planning no matter the scale of production or commodity can all prove to be significant steps towards shifting farmer’s minds and encouraging capacity development for climate-smart practices and innovations.

## Implications of climate variability to the Ncunjane community

Ncunjane represents a stable community bound to traditional practices evident in keeping large cattle herds, for cultural slaughter and draught power and having many generational families. People live in compound homesteads with extended family members and because polygamy is practiced, there are generally two or more wives, *omakoti*, found living in a single homestead. Ncunjane formed one of the four land purchases in July 1996, which marked the first land transfer, under the Land Reform Pilot Programme. The process was facilitated by district planners (Greenberg 1996:89). Transfer of title deeds for properties Springs 13210 (Vernier) and Koorn Spruit 4355 (Aston Lodge) occurred in 2000 (Umtshezi Local Municipality 2012).

The area is suited to extensive farming systems with a low potential for production and most areas being declared non-arable because of a high risk of erosion with an average rainfall of 601 mm – 700 mm per annum (Umtshezi Local Municipality 2012). Agricultural potential, within the district, allows for mixed crop and livestock farming systems, mainly goat production, to take place. The most significant irrigation takes place in the valley of the Bushman’s River, which boasts good potential land situated along the Tugela River and around the Weenen town. In areas further from the floodplains, crops are cultivated under dry land agriculture and have relatively lower yields as compared to crops under irrigation (Umtshezi Local Municipality 2012).

### Climate change poses a threat to agriculture as a feasible economic livelihood source for rural households

Severe drought and on-going dry spells including excessive heat waves pose the most threat to dry land agriculture in the area. Producers may have to start asking tough questions about their future participation in the local agricultural value chain. ‘Stepping up’ or ‘just hanging on’ are concepts that will require serious evaluation if the sustainability of the agriculture as a major livelihood source is still relevant in the next decade to come. Households may have to look to alternative livelihood sources, which are less affected by changes in climate. On the other hand, households aspiring to re-enter into agriculture will have to conduct watertight business plans backed by feasibility studies and a bucket full of prayers to the rain gods. Drawing from the Ncunjane case study, vegetable and maize production provided households with constant supplies of food, thus relieving pressure off already limited disposable income so that other household needs are met. With the agricultural production decline looming, households will have to further stretch their constrained budgets to buy food items such as vegetables and maize, a key ingredient in traditional home brew and other traditional recipes. The effects of exiting from agriculture because of excessive climate change impact and increased vulnerability will strike a huge blow to rural households that have historically relied on crop and animal production for a huge proportion of their food consumption. Culture and tradition will equally take a huge blow. As such, many households engaged in mixed-farming systems will be at the crossroads having to take tough decisions on whether available limited resources will be prioritised for either crop or livestock production systems or neither.

### Overstretched constrained government drought relief support

Government drought relief support to smallholder producers is often very limited with the impact being far below expectations as government funds are overstretched given the large number of producers affected by the drought. At times, the support comes a little too late. Rukema (2013) who also investigated drought and people’s resilience in Msinga proposed that the *Disaster Management Act* of 2002 should ensure that any form of financial and bureaucratic bottlenecks are eliminated to maximise programme outreach in a timely manner. The development of long-term objectives that will enhance the adaptive capacity of farming communities like Ncunjane should also be on the agenda of the state (Rukema 2013). It is also important that non-government organisations and community-based organisations in the area are empowered and involved in the design of disaster risk management plans. Private sector role players need to actively leverage government funding for the implementation of drought relief support programmes.

### Threatened water supplies further limit irrigation potential

The potential role of introducing irrigation as a mitigation measure under dry land agriculture could boost productivity and reduce livestock mortality. However, prolonged drought spells and high evapotranspiration has left dams and rivers dry. Access to borehole water would be more feasible pending availability of groundwater and diesel to operate the pump. Apportioning water for agriculture versus domestic use is proving to be a tug-of-war as the only water source available during drought is through the municipal subsidised water tankers. Therefore, prospects for graduation from smallholder agriculture are highly compromised because of erratic rainfall and lack of irrigation infrastructure.

### Adapt or die: Innovation for adaptation of smallholder mixed production systems

Animal production serves various cultural purposes and signifies men’s socio-economic status within the rural setting, which makes reducing cattle and goat numbers a challenge. During the drought incident in 2010, households experienced high cattle losses simply from lack of water and supplementary feed. During the 2014–2015 drought incident, these households fell victim to the same fate. Should the status quo persist without adopting robust alternatives to adapt during drought or other likely extreme climatic events, practices such as cultural rituals requiring sacrifice of animals, payment of dowry for sons who would carry through the family name are among many functions served by livestock that are at risk with the awakening of climate change.

The draft Policy on Conservation Agriculture (Department of Agriculture Forestry and Fisheries 2016) recognises that the current farming methods have a high environmental demand and are largely dependent on external inputs, thus leading to the depletion and degradation of natural resources. Le Roux (2014) shares these sentiments by pointing out farming practices and agricultural intensification as the main culprits for major soil erosion. He presents recent soil erosion maps, which position the KZN Province as the third (87 522 ha) and fifth (1 284 975 ha) province most affected by gully and sheet and rill erosion, respectively. This is most prevalent in annual croplands (grain crops), where soil erosion occurs at an estimated rate of 13 tons/ha/year, which was found to be higher than the natural soil formation rate of 5 tons/ha/year. Essentially, these findings imply that we lose more soil than we gain.

Moreover, other scientific data corroborate significant correlation between soil erosion and increased costs of food production. They reverberate the call for conservation agriculture as a crucial mitigation measure to be put in place as it has multiple benefits for the soil (Le Roux 2014; Ngaka 2012). For instance, the use of cover crops and retaining crop residues on the soil surface were found to promote a healthy strong root system, which greatly enhanced resistance to soil erosion. Rukema (2013) emphatically advocates for the inculcation of indigenous knowledge systems in the construction of community-based coping strategies stating that the utility value of these knowledge systems has stood the test of time and they are well understood by the people who practice them. In light of this perspective, the culling of livestock numbers per household through auction sales is encouraged to guard against high cattle mortality rates during drought.

## Conclusion and recommendations

The article has attempted to unpack key concepts of climate change and has shown how they were applied in the study area. The effects of climate change to the Ncunjane community were discussed together with some of the factors that can either increase vulnerability or increase adaptation. The Ncunjane farming community has been found to have a poorly structured risk mitigation plan for livestock as there is a high dependency on government drought relief support among livestock owners. Extreme heat spells were found to lead to a complete halt of cropping activities, which put pressure on household disposable income given rising food prices. The lack of alternative irrigation water sources poses a major threat to the sustainability of agriculture, and should this persist, households will have to step out of agriculture in search of more reliable livelihoods sources. However, localised innovative strategies that rely on indigenous knowledge systems can contribute to improving the adaptive capacity of these farming households.

It is clear that climate variability and social vulnerability are interrelated concepts that need to be understood better as this can begin to guide planning for increasing adaptation of communities to current and future extreme scenarios. Based on the findings of the study, the following recommendations are made:

- Construction of an average-size dam by the local municipality for both domestic and irrigation purposes.
- Incentivise producers who apply innovation in areas such as water-use management and climate-smart agriculture.
- Consider the adoption of a combination of strategies based on indigenous knowledge systems and bioresource management techniques through the application of alternative methods such as water harvesting; use of grey water; mulching and deep-trench beds for vegetable production.
- Implement climate-smart innovations such as the used of advanced or hybrid cultivars (drought-resistant crop or grass varieties) and hardy cattle breeds and goat production.
- Consider agroecological principles such as agroforestry, conservation agriculture or climate-smart agriculture.

More strategic policy–related and programme-related recommendations include the following:

- Train public extension and advisory services, animal health practitioners including social workers on climate change and adaption by society from a social, ecological and economic perspective. This calls for investment in employee capacity building and equipping them with the appropriate working tools or resources.
- Government to promote climate-smart innovations such as greenhouses and hydroponics through subsidies and low-interest loan facilities.
- Strengthen institutional mechanisms and promote intergovernmental relations for successful implementation of government drought relief support programmes.
- Encourage public–private partnerships to leverage support for producers affected by climate change or drought.
- Research institutions to prioritise climate-smart innovations.
- Strengthen use of ICT and social networks through increased investment in rural ICT infrastructure, broadband and development of affordable, user-friendly applications.

Adaptation presents producers with prospects for growth within agriculture in the awakening impact of climate change given that over time producers will eventually fully comprehend climate change and begin thriving in the advantages that comes with it.
